# Prevalence and associated factors with oral frailty in middle-aged and older hospitalized patients: a cross-sectional study

**DOI:** 10.3389/fpubh.2025.1446862

**Published:** 2025-02-18

**Authors:** Jun-kai Dou, Huan Liu, Jiahui Min, Yang Luo, Qian Liu, Xue-zhi Shi, Xiubin Tao, Ming Zhang

**Affiliations:** ^1^Nursing Department, Lu’an Hospital of Anhui Medical University, Lu'an, Anhui, China; ^2^Nursing of Hemodialysis, The First Affiliated Hospital of Wannan Medical College (Yijishan Hospital of Wannan Medical College), Wuhu, Anhui, China; ^3^School of Public Health, Wannan Medical College, Wuhu, Anhui, China; ^4^School of Clinical Medical, Wannan Medical College, Wuhu, Anhui, China; ^5^School of Stomatology, Wannan Medical College, Wuhu, Anhui, China; ^6^Department of Nursing, The First Affiliated Hospital of Wannan Medical College (Yijishan Hospital of Wannan Medical College), Wuhu, Anhui, China; ^7^School of Educational Science, Anhui Normal University, Wuhu, Anhui, China; ^8^School of Innovation and Entrepreneurship, Wannan Medical College, Wuhu, Anhui, China

**Keywords:** comorbidities, frailty, social frailty, cognitive, medication compliance

## Abstract

**Background:**

With the development of the economy and society, people pay more and more attention to oral health. Oral frailty can limit nutritional intake and make an individual physically weak, which is detrimental to people’s health. Therefore, it is urgent to identify oral frailty and their associated risk factors. In this study, we aimed to evaluate the oral frailty and its influencing factors in Chinese middle-aged and older hospitalized patients.

**Methods:**

A cross-sectional study was conducted from May 2023 to February 2024 in 2 tertiary hospitals in Wuhu City, Anhui Province, China. The self-designed sociodemographic information, the Oral Frailty Index-8, the 2-item Connor–Davidson resilience scale (CD-RISC-2), the sarcopenia screening questionnaire, and the three-item short literacy survey were used in this study.

**Results:**

A total of 914 middle-aged and older adults patients were recruited. The prevalence of oral frailty was 48.7% (445/914). In the univariate analysis, oral frailty was significantly associated with age, education, place of residence, monthly income, sarcopenia, resilience, and health literacy. Correlation analysis showed that oral frailty was significantly negatively correlated with sarcopenia, resilience, and health literacy. In the binary logistic regression analysis, oral frailty was significantly associated with sarcopenia, and health literacy. Health literacy was a protective factor of oral frailty.

**Conclusion:**

This study aimed to investigate the prevalence of oral frailty and identify the associated influencing factors among middle-aged and older adults patients in Anhui Province, China. This study identified several factors influencing oral frailty in middle-aged and older adults patients. Therefore, the government and relevant departments should implement targeted interventions to improve middle-aged and older adults patients’ oral frailty.

## Introduction

In recent decades, life expectancy has increased worldwide and the proportion of middle-aged and older adults people in other age groups has continued to increase, resulting in an aging society. As China’s aging population intensifies and lifestyle changes, the disease problems of middle-aged and older adults people have received widespread attention from society. The Japanese Dental Association defines oral frailty as a series of age-related phenomena and processes that lead to changes in various oral conditions (number of teeth, oral hygiene, oral function, etc.) and are accompanied by a decrease in interest in a healthy oral cavity. It is defined as decreased physical and mental performance, oral weakness leading to weight gain and eating disorders. The overall impact is reduced physical and mental performance (1). Oral health is one of the important components that affect the high quality of life of the older adults (1). Oral health is essential in maintaining good general health (2).

Studies have shown that poor oral health is associated with age-related physiological stress, frailty (3), sarcopenia (4), cognitive decline (5), and the accumulation of multiple diseases (6). Over and above all, oral frailty increases the risk of hospitalization for illness in middle-aged and older adults. People have recently become aware of the risk of periodontal disease in patients with chronic diseases such as diabetes (7), coronary heart disease (8), respiratory disease (9), and osteoporosis (10). Oral frailty may be an essential risk factor for systemic diseases, so timely oral health management is crucial to prevent non-communicable chronic diseases (11). A study found that people with oral frailty were at higher risk for physical frailty, sarcopenia, serious illness requiring care, and death than people without oral weakness (12). Poor oral health could affect the ability to eat, taste, swallow, and speak, reduce self-confidence, and significantly impact quality of life (13). Hihara et al. (14) assumed that oral frailty was an early sign of physical frailty.

Sarcopenia is a pathological condition of “muscle failure,” defined as “the progressive loss of muscle strength (muscle deficiency), mass (quantity), and function (quality), resulting in the decreased physical performance and increased disability, risk of falls, and death Rate (15). Sarcopenia is a multifaceted age-related disorder involving biological, environmental, socioeconomic, and genetic factors that combine to cause loss of muscle mass and function (16). Sarcopenia is an essential symbol of the aging process and is closely related to adverse outcomes such as falls, disability, and death, seriously affecting the physical health and quality of life of middle-aged and older adults people. The development of sarcopenia is related to unique molecular mechanisms such as aging, chronic disease, malnutrition, and inactivity (16). Sarcopenia is a reversible disease that can be reversed with early intervention (17).

Rutter (18) proposed psychological resilience in 1987 and defined it as the positive impact of individual differences on responses to stress and adversity. Psychological resilience can affect people’s emotions directly or indirectly through reactions. The study proposed that greater resilience is related to better-coping behaviors, and coping behaviors are related to better cognitive functions (19). At the same time, psychological resilience could protect against the harmful effects of chronic stress (20). Resilience is a dynamic process that allows people with chronic illness to adapt more positively when faced with unpleasant experiences in their lives, which allows them to manage better situations that cause anxiety and stress (21). Resilience can improve a person’s self-esteem, personal strength, and emotional stability. People with high levels of resilience have an extraordinary ability to cope with disease-related challenges (22).

Health literacy (HL) is a set of personal skills for acquiring, processing, understanding, and using health information to maintain health (23). Health literacy is essential for healthy living but is especially important when making disease-related decisions. These skills will help people prevent illness but also help people make informed decisions when they become ill, allowing them to manage their illness and the self-care that comes with it. Factors associated with health literacy include patient characteristics (24), physician-patient communication (25), the way information is presented ((26), and the way an individual receives information about their disease (also known as organizational HL). People with sufficient HL can identify and access reliable sources of information, distinguish misinformation, identify risks, and analyze data about their situation (27). In general, adequate HL relates to the patient’s ability to manage disease, communicate effectively with healthcare professionals, and participate in medical decision-making (28). Therefore, appropriate LH can guide and ultimately help patients on the right treatment path.

Oral frailty is an important feature of unhealthy aging, with serious negative effects on quality of life and an increased risk of malnutrition and death in middle-aged and older hospitalized patients. Early intervention and improvement of oral health are critical to the physical health of middle-aged and older adults and to reducing future adverse health events. Exploring the current status and influencing factors of oral frailty in middle-aged and older adults people, as well as factors that prevent the negative effects of oral frailty (e.g., resilience and social support), are essential for developing future guidelines for the prevention of oral frailty. However, the prevalence data of oral frailty in middle-aged and older hospitalized patients with chronic diseases in China are still lacking. Therefore, it is of great academic and practical significance to explore the status quo of oral frailty and related influencing factors in middle-aged and older hospitalized patients. The purpose of this study is to enrich the research on oral frailty by analyzing the prevalence and related influencing factors of oral frailty in middle-aged and older hospitalized patients in Anhui province. The results of this study can provide reference for medical institutions to improve the quality of life of middle-aged and older hospitalized patients with oral frailty in Anhui province.

## Materials and methods

### Setting and participants

All investigators composed of undergraduate students from Wannan Medical College, and they received systematic training before the start of the survey to improve the scientific nature of the investigation and questioning skills, and reduce investigation errors. When the patient’s vital signs and conditions were relatively stable, the researcher carefully explained the purpose of this study to middle-aged and older adults patients with chronic diseases who met the inclusion criteria. Our investigators, supported by hospital nursing staff, conducted face-to-face interviews with participants using a structured questionnaire. Verbal informed consent was obtained from all participants. After excluding patients with extremely serious conditions and those who could not fully understand the oral explanation, 981 valid samples were finally included. The investigators fully ensured participant’s privacy, confidentiality, voluntary participation, and their unconditional right to withdraw from the study at any point of data collection without causing any harm. This was a multicenter cross-sectional study, and participants were recruited from 2 tertiary hospitals in Wuhu City using convenience sampling. The inclusion criteria are: (1) age ≥ 45 years or over, (2) Chronic diseases such as diabetes, hypertension, stroke, and coronary heart disease were diagnosed in secondary hospitals and above, (3) Participants who have the ability to complete the questionnaire independently or can do so with the face-to-face assistance of the investigator.

The exclusion criteria are (1) the participants were diagnosed with severe mental illnesses, and neurological diseases by professional medical institutions; (2) those with a certain degree of cognitive impairment and unable to complete the questionnaire; (3) middle-aged and older adults patients with severe cardiovascular and cerebrovascular diseases, severe liver and kidney diseases, malignant tumors and other end-stage diseases; (4) severe vision or hearing impairment, unable to cooperate in completing the questionnaire.

### Measurements

#### Sociodemographic variables

The general demographic characteristics questionnaire was developed by searching the relevant literature through databases such as web of science and Pubmed and the clinical experience of the researchers.

#### Oral Frailty Index-8 (OFI-8)

This scale was compiled by Tanaka et al. (29), and was used to screen patients for oral frailty. The scale includes 5 aspects: whether to use dentures, swallowing function, social participation, oral health-related behaviors and chewing ability, with a total of 8 items, The OFI-8 total score was 0–8 points, and the higher the score, the worse the oral frailty, with ≥4 points indicating oral frailty. Moreover, its effectiveness has been verified through multiple cross-sectional surveys (30). The scale has good reliability and validity, with Cronbach’s alpha coefficient is 0.84 in this study.

#### The CD-RISC-2

The 2-item Connor–Davidson resilience scale (CD-RISC-2) is a self-assessment tool used to measure psychological resilience (31). The CD-RISC-2 consists of 2 questions, questions include “I can adapt when people and things around me change” and the responses are on a five-point Likert scale ranging from 0 (“never”) to 4 (“always”), generating total scores from 0 to 8, with higher scores indicating more higher degree of psychological resilience. The Cronbach’s alpha of the scale was 0.82 in this study.

#### SARC-F questionnaire

Sarcopenia was assessed using the Sarcopenia Screening Questionnaire developed by Saint Louis University in the United States (32). It is currently widely used in many clinical studies because it is easy to operate and does not require other auxiliary equipment. SARC-F questionnaire components five components: strength, assistance walking, rise from a chair, climb stairs, and falls. Each item ranges between 0 and 2, and the total score ranges between 0 and 10.The higher the total SARC-F score, the more severe the sarcopenia. A score of 4 or greater predicts sarcopenia. The Cronbach’s alpha of the scale was 0.83 in this study.

#### Health literacy

The “Three-item Short Literacy Survey” scale was used. It was verified by McNaughton et al. (33) that this form has the characteristics of simplicity, speed, and good reliability and validity. This form is suitable for the health literacy assessment of middle-aged and older adults inpatients. The content includes: (1) Do you often need someone (such as family, friends, medical workers, caregivers, etc.) to help you understand written medical information? (2) Do you feel confident filling out medical forms on your own? (3) Do you need help interpreting written medical information to better understand your medical condition?”. The answers to each question are divided into 5 levels, each assigned a score of 1–5, with a total score of 15. A score of ≤10 indicates health literacy, and a score of >10 indicates a lack of health literacy. The Cronbach’s *α* coefficient is 0.792.

### Statistical analysis

All the collected data were stored in the Questionnaire Star database and later imported into the Statistical Package for Social Sciences (SPSS) V22.0 software for data analysis. The participants’ oral frailty status was classified as oral frailty or non-oral frailty. Data were expressed as means and standard deviations or frequencies and percentages, and *χ*^2^ tests were performed to assess the relationships between factors and outcomes. Chi-square test was used to identify statistically significant factors affecting oral frailty. Independent variables with *p*-values less than 0.05 were used in binary regression analysis to identify variables which were independently associated with oral frailty. All statistical analyses were two-tailed, and statistical significance was set at *p* < 0.05 (Two-tailed).

### Ethical consideration

This study was approved by the Nursing Department of the First Affiliated Hospital of Wannan Medical College (Yijishan Hospital), China (YJYYYHLB-2023-09-07). The survey was in line with the principles outlined in the latest edition of the Declaration of Helsinki. Participation in the study was voluntary, and all participants received verbal informed consent and signed a paper informed consent form at the beginning of the study.

## Results

### Participant characteristics

A total of 943 middle-aged and older adults patients were invited to participate this study, of which 29 participants were excluded due to incomplete answer. The final sample recruited 914 patients, with the mean age being (65.73 ± 10.56) years old, of whom 312 (34.1%) patients aged between 45 and 59 years old, 246 (26.9%) patients aged between 60 and 69 years old, 250 (27.4%) patients aged between 70 and 79 years old, and 106 (11.6%) patients aged 80 years or older. 527 (57.5%) were male, and 388 (42.5%) were female. 316 (34.6%) patients resided in countryside, 332 (36.3%) resided in town, and 266 (29.1%) resided in city. The socio-demographic information is showed in Table 1.

**Table 1:** Participants’ demographic information (*N* = 914).

Variable	Category	Participants	Percentage (%)
Gender	Male	526	57.5
	Female	388	42.5
Age	45–59	312	34.1
	60–69	246	26.9
	70–79	250	27.4
	≥80	106	11.6
Place of residence	Countryside	316	34.6
	Town	332	36.3
	City	266	29.1
Education	Elementary school and below	527	57.7
	Junior school	250	27.4
	High school	93	10.2
	Technical secondary school	26	2.7
	College and above	18	2.0
Monthly income	<1,000	249	27.2
	1,000–2000	319	34.9
	2001–3,000	166	18.2
	3,001–4,000	102	11.2
	4,001–5,000	45	4.9
	>5,000	33	3.6
Medical insurance type	Employee	169	18.5
	Resident	160	17.5
	New rural cooperative	527	57.7
	Other	58	6.3
Regular physical examination	No	486	53.2
	Yes	428	46.8
Oral cleaning after eating	No	519	56.8
	Yes	395	43.2

### Univariate analysis of risk factors for oral frailty in middle-aged and older adults patients

In this study, 445 (48.7%) middle aged and older adults patients were identified as oral frailty. The result of univariate analysis showed that age, education, place of residence, monthly income, sarcopenia, resilience, and health literacy were inverse (Table 2).

**Table 2:** Characteristics of the participants based on the presence of oral frailty (*N* = 914).

	Non-OF	OF	*χ^2^*	*P*
Gender			3.561	0.059
Male	284(60.6%)	242(54.4%)		
Female	185(39.4%)	203(45.6%)		
Age			101.124	<0.001
45–59	217(69.6%)	95(30.4%)		
60–69	138(56.1%)	108(43.9%)		
70–79	89(35.6%)	161(64.4%)		
≥80	25(23.6%)	81(76.4%)		
Education			9.949	0.041
Elementary school and below	247(46.9%)	280(53.1%)		
Junior school	143(57.2%)	107(42.8%)		
High school	53(57.0%)	40(43.0%)		
Technical secondary school	15(57.7%)	11(42.3%)		
College and above	11(61.1%)	7(38.9%)		
Place of residence			6.562	0.038
Countryside	162(51.3%)	154(34.6%)		
Town	186(56.0%)	146(44.0%)		
City	121(45.5%)	145(54.5%)		
Monthly income			30.098	<0.001
<1,000	98(39.4%)	151(33.9%)		
1,000–2000	162(50.8%)	157(49.2%)		
2001–3,000	96(57.8%)	70(42.2%)		
3,001–4,000	70(68.6%)	32(31.4%)		
4,001–5,000	26(57.8%)	19(42.2%)		
>5,000	17(51.5%)	16(48.5%)		
Medical insurance type			0.763	0.858
Employee	85(50.3%)	84(49.7%)		
Resident	87(54.4%)	73(45.6%)		
New rural cooperative	267(50.7%)	260(58.4%)		
Other	30(51.7%)	28(48.3%)		
Regular physical examination			0.100	0.752
No	247(50.8%)	239(49.2%)		
Yes	222(51.9%)	206(46.3%)		
Oral cleaning after eating			0.129	0.720
No	200(50.6%)	195(49.4%)		
Yes	269(51.8%)	250(48.2%)		
Sarcopenia			71.903	<0.001
No	428(58.2%)	307(41.8%)		
Yes	41(22.9%)	138(77.1%)		
Resilience	8.0(7.0,9.0)	7.0(6.0,7.0)	−8.386	0.001
Health literacy	8.0(6.0,10.0)	7.0(4.0,9.0)	−4.501	<0.001

### Correlations between oral frailty and relevant indicators

As shown in Table 3, sarcopenia had positive correlation with oral frailty (*p* < 0.01), in contrast, resilience and health literacy had negative correlations (*p* < 0.01).

**Table 3:** Correlation between oral frailty and relevant indicators in middle-aged and older adults patients.

Factors	*r*	*p-*value
Sarcopenia	0.458	<0.001**
Resilience	−0.235	<0.001**
Health literacy	−0.231	<0.001**

**p < 0.001.

### Binary analysis of influencing factors of oral frailty

**Figure 1:**
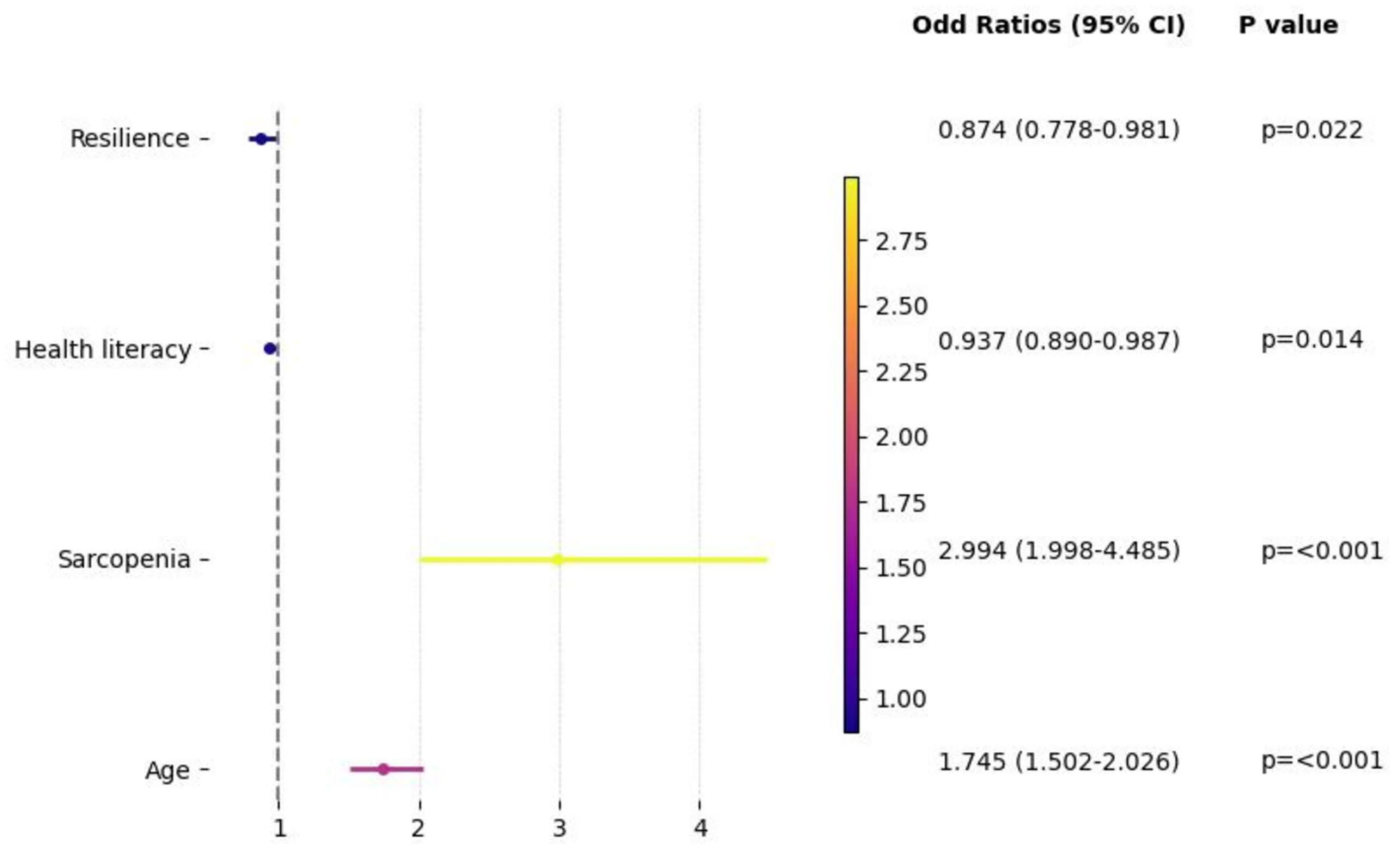
Forest plot: factors affecting oral frailty using binary logistic regression analysis.

Binary logistic regression analysis was performed to explore the influencing factors of oral frailty, in which age, education, place of residence, monthly income, sarcopenia, resilience and health literacy were used as independent variables and oral frailty (grouping, 0 = non-oral frailty, 1 = oral frailty) were used as dependent variables. The result displayed that middle-aged and older adults patients with the age increasing, the risk of oral frailty increased (OR = 1.745, 95% CI 1.502–2.026). Compared with non-sarcopenia middle-aged and older adults patients, patients with sarcopenia more likely to experience oral frailty (OR = 2.994, 95% CI 1.998–4.485). Besides, our findings showed that health literacy (OR = 0.937, 95% CI 0.890–0.987) and resilience (OR = 0.874, 95% CI 0.778–0.981) were protective factors for oral frailty among middle-aged and older adults patients. The result of binary logistic regression analysis is displayed in Figure 1 and Table 4.

**Table 4:** Binary logistic regression analysis of factors associated with oral frailty.

Indices	*β*	Wald	*p-*value	OR	95% CI
Age	0.557	53.178	<0.001	1.745	1.502–2.026
Sarcopenia (No = 0, Yes = 1)	1.097	28.275	<0.001	2.994	1.998–4.485
Health literacy	−0.065	6.042	0.014	0.937	0.890–0.987
Resilience	−0.135	5.263	0.022	0.874	0.778–0.981

## Discussion

With the aging process, middle-aged and older populations are increasingly susceptible to oral frailty, which manifests as tooth loss, poor oral hygiene, and swallowing difficulties. These issues contribute to the deterioration of general health. While extensive research has examined oral frailty in community-dwelling older adults, there is a significant research gap regarding hospitalized middle-aged and older patients in China. This limitation hinders medical professionals in detecting and implementing effective preventive measures against oral frailty. To address this gap, our study utilized the Oral Frailty Index-8 questionnaire to investigate the prevalence and influencing factors of oral frailty among hospitalized patients.

Our findings revealed that 48.7% of patients aged 45 years and older in China are at risk of oral frailty, a prevalence significantly higher than the annual incidence reported among community-dwelling Chinese older adults (30.3%) (34). This discrepancy likely arises from the higher prevalence of physical frailty, sarcopenia, and malnutrition among hospitalized patients (35), which may lead them to rate their oral health more poorly. These results underscore the need for targeted interventions for this vulnerable population.

The study confirmed that advancing age is a critical risk factor for oral frailty, consistent with previous research by Iwasaki et al. (36). Age-related factors such as increased tooth loss, reliance on dentures, reduced diet quality, and difficulties in eating or chewing solid foods are well-documented contributors to poor oral health (37, 38). Additionally, older adults in China exhibit low levels of oral hygiene and infrequent dental visits (39), exacerbating the risk of oral frailty. Furthermore, advancing age is often accompanied by physical and social frailty as well as reduced social engagement, all of which elevate the risk of oral frailty (40). These findings highlight the importance of implementing standardized oral frailty screening during hospital admissions and initiating preventative measures, particularly for older patients.

Our study also identified sarcopenia as a significant risk factor for oral frailty in middle-aged and older patients, aligning with previous research (12). Sarcopenia, an age-related syndrome, adversely affects occlusal force (41), masticatory ability (42), tongue pressure (43), and swallowing function (44), resulting in diminished oral functionality. Kugimiya et al. (45) demonstrated that older adults with sarcopenia are more likely to exhibit low oral function. This emphasizes the need for patients with sarcopenia to receive regular dental care and targeted interventions to maintain oral functionality.

Additionally, we found that patients with higher health literacy scores were more likely to experience oral frailty. Although health literacy is critical for disease management, self-care, and the effective use of preventive services (46), its role in oral frailty has not been previously studied. Given that health literacy is vital in preventing general frailty (47), it may also play a key role in improving and preventing oral frailty. Future studies should explore the mechanisms underlying this relationship to inform interventions aimed at improving oral health outcomes.

Lastly, our results revealed a positive relationship between resilience and oral frailty. Resilience, defined as a reserve capacity to overcome stress and adversity (48), has been shown to promote healthy behaviors and mitigate the impact of disease (49). However, a high level of resilience has also been associated with poor self-rated oral health and oral health problems (50), possibly due to a greater awareness of health issues. Previous studies have suggested that resilience mediates the relationship between depression and oral health (51). These findings suggest that enhancing resilience may be beneficial for addressing poor oral health, and targeted psychosocial interventions should be considered.

This study highlights the high prevalence of oral frailty among hospitalized middle-aged and older patients in China and identifies key risk factors, including advancing age, sarcopenia, health literacy, and resilience. These findings underscore the importance of standardized oral frailty screening and targeted interventions to improve oral health outcomes in this vulnerable population. Future research should further investigate the mechanisms linking these factors to oral frailty to develop evidence-based strategies for prevention and management.

### Limitations

The current study has several limitations concerning the generalizability of the results: first, this study is cross-sectional and cannot infer causal relationships between influencing factors and oral frailty. Secondly, this study was conducted only in two tertiary hospitals in Wuhu City, Anhui Province, and this geographical limitation reduces the external validity of the study results. Third, self-report survey methods are susceptible to subject recall bias and social desirability bias, which may affect the accuracy of reported information. Fourth, potential sampling bias is also an unavoidable limitation. Therefore, this study will further expand the scope of the survey and conduct a series of longitudinal surveys in the future to verify and expand the relevant results.

## Conclusion

The prevalence of oral frailty among middle-aged and older hospitalized patients was notably higher compared to community-dwelling older adults, indicating a critical need for targeted interventions in this population. Age and sarcopenia were identified as significant risk factors for oral frailty, while health literacy and resilience emerged as protective factors. These findings underscore the importance of routine screening for oral frailty in hospitalized patients, particularly those at higher risk, such as individuals with sarcopenia, low health literacy, low resilience, and advanced age. Health care professionals should implement comprehensive programs to identify and manage oral frailty early, incorporating strategies to enhance health literacy, improve resilience, and address the underlying causes of sarcopenia. Such measures are essential to mitigate the progression of oral frailty and improve overall patient outcomes.

## Data Availability

The raw data supporting the conclusions of this article will be made available by the authors, without undue reservation.
